# Surgical Treatments for Women with Stress Urinary Incontinence: A Systematic Review

**DOI:** 10.3390/life13071480

**Published:** 2023-06-30

**Authors:** Bogdan Cristian Călinescu, Adrian Neacșu, Ana Elena Martiniuc, Dan Dumitrescu, Catalina Diana Stănică, George-Alexandru Roșu, Laura Ioana Chivu, Raluca Gabriela Ioan

**Affiliations:** 1 Department of Obstetrics-Gynecology, INSMC Alessandrescu Russescu, 38-52 Gheorghe Polizu Str., 127715 Bucharest, Romania; bogdan-cristian.calinescu@drd.umfcd.ro (B.C.C.); raluca.ioan@umfcd.ro (R.G.I.); 2 Department of Obstetrics-Gynecology and Neonatology, University of Medicine and Pharmacy “Carol Davila”, 37 Dionisie Lupu Str., 020021 Bucharest, Romania; adrianneacsu2006@yahoo.com (A.N.); anilenac@yahoo.com (A.E.M.); catalina.stanica@umfcd.ro (C.D.S.); 3 Department of General Surgery, Emergency University Hospital, University of Medicine and Pharmacy “Carol Davila”, 168 Splaiul Independentei Str., 050098 Bucharest, Romania; dan.dumitrescu@umfcd.ro; 4 Department of Obstetrics-Gynecology and Neonatology, Saint Pantelimon Emergency Hospital, University of Medicine and Pharmacy “Carol Davila”, 340-342 Pantelimon Str., 021659 Bucharest, Romania; george.rosu@umfcd.ro; 5 Department of Pathophysiology, University of Medicine and Pharmacy “Carol Davila”, 050474 Bucharest, Romania

**Keywords:** stress urinary incontinence, Burch surgery, midurethral sling, pubovaginal sling

## Abstract

Stress urinary incontinence affects a large proportion of women in their lifetime. The objective of this review was to describe and compare the latest surgical trends in urinary incontinence and focus on the literature advantages, disadvantages, complications and efficacy of surgical procedures regarding this pathology. Using network meta-analysis, we have identified the most frequently used procedures (Burch surgery, midurethral sling and pubovaginal sling), and we have described and characterized them in terms of effectiveness and safety. Midurethral procedures remain the gold standard for surgical treatment of stress urinary incontinence, although the potential of serious complications following this procedure should be taken into consideration always. There is a clear need for a much more unified evaluation of possible complications and postoperative evolution. This process will help practitioners to adapt and individualize their strategy for each patient.

## 1. Introduction

Urinary incontinence is defined by the International Continence Association as any involuntary leakage of urine. Most of the time, it occurs due to physical activity that puts pressure on the bladder, such as exercise, sneezing, coughing, laughing or bending over [1,2].

All over the world, it is a worrying condition that can reduce the quality of life in women. Furthermore, the economic burden is considered to be high. The prevalence varies from 20% to 50% throughout life but is seen more often in women who have had children, and it becomes higher with age [1,2]. There are several types of urinary incontinence, including stress incontinence, urge incontinence, overflow incontinence (chronic urinary retention) or total incontinence. Each of these types has its own causes, but most of the time, the causes may be common. Some of the possible causes lead to short-term urinary incontinence, while others may cause long-term problems [3].

In Europe, the prevalence of SUI was estimated in 2022 at 14.5% according to a study carried out in 2022 in the population group of 30–60 years, and in Romania, it is evaluated to be 18%, maybe even higher due to the poor addressability of the patient regarding this pathology [4]. 

Stress incontinence appears when the pressure inside the bladder, as it fills with urine, becomes greater than the strength of the urethra to stay closed [3]. 

If the muscles that keep the urethra closed are damaged, the urethra may not be able to stay continent. Problems with the pelvic floor muscles may be caused by childbirth, increased pressure in the abdomen, surgery in the pelvic area, connective tissue disorders or neurological conditions [3].

Although urinary incontinence is a non-life-threatening condition, it is known to have a significant health impact on the aging female population and leads to low quality of life in many ways. When conservative treatments have failed to control the condition, surgical treatment is necessary.

Surgical treatment for stress urinary incontinence was described with a lot of procedures, each new procedure was developed to overcome the previous ones, but this situation led to a lot of ambiguities [5].

In 1998, a prospective study was conducted on the comparison between the medium- and long-term effects of various surgical treatment procedures in female stress urinary incontinence. Basically, at that time, three types of surgical interventions were performed: the Kelly suburethral plication, transvaginal needle suspension (or its variants) and Burch colposuspension [5,6,7].

In the beginning, the indications for one of the procedures depended on the surgeon’s choice, the Kelly plication procedure being predominant due to its technical simplicity and simple postoperative evolution. This approach seemed to be easy and a little time-consuming from the technical perspective than the other two procedures and, for a long time, was the standard surgical procedure for stress urinary incontinence [7,8].

These interventions were surpassed by midurethral sling surgery developed in the 1990s, described as a minimaly invasive surgery that used a strip of synthetic mesh (sling) that was placed with no fixation (tension-free) on the structures that needed to be suspended [6,8].

Until the development of urodynamic investigation equipment (1998), the selection criteria for the procedures were exclusively clinical and limited to the recurrent form, especially after the Kelly operation, or to certain forms considered to be more complicated. The introduction of a urodynamic investigation managed, first of all, to objectify the type of incontinence preoperatively and objectively analyze a segment of the operated patients. Many patients did not find themselves in a subsequent segment because of the high subjectivity regarding self-evaluation. Moreover, a large number of recurrences were not found in the computerized statistics of the urodynamic apparatus [8,9]. 

Currently, there are different types of surgical treatments for this condition, including the following:

- Anterior vaginal repair colporrhaphy (anterior repair);
- Bladder neck needle suspensions;
- Open retropubic abdominal colposuspension;
- Retrosuspension made laparoscopic;
- Traditional sub-urethral retropubic sling;
- Midurethral retropubic sling (retro-MUS);
- Transobturator midurethral sling (transob-MUS);
- Single-incision slings small procedures;
- Peri-urethral injectable bulking agents.

Each of these operations can be performed with different techniques, and each has its own advantages and disadvantages. Because of the lack of consensus and the different types and techniques used to perform this surgical operation, it is difficult to determine which procedure should be better used [10]. Comparative safety profile of these surgical procedures is still unclear because of the lack of long-term evidence in most of the studies [10]. There is a clear need to declare adverse events after surgery for stress urinary incontinence and a personalized strategy based on women’s symptomology, medical comorbidities, and intra-operative risk factors. A personalized strategy should always be the best strategy in the treatment of each of the cases [11].

In this paper, we describe the latest trends in SUI with a focus on the literature advantages, disadvantages, complications and efficacy. Besides the description of the techniques, their analysis and comparison are extremely useful for refining the selection process of an individualized technique for each patient.

The motivation for this review comes from the need to increase the addressability of this pathology with multiple personal, social and economic implications. At this moment, there is a multitude of surgical techniques described, each with its advantages and disadvantages, but there is a clear need for a much more unified and anatomically adapted approach to this problem. The multitude of techniques described leads to a non-unitary evaluation of possible complications and postoperative evolution. The purpose of the review was to draw attention to these ambiguities and to establish the clear need for collaboration in order to standardize procedures and declare complications.

Standardization of interventions, as well as a more accurate declaration of existing postoperative complications, would help practitioners to adapt and individualize their strategy.

### 1.1. Search Strategy and Data Synthesis

**Figure 1:**
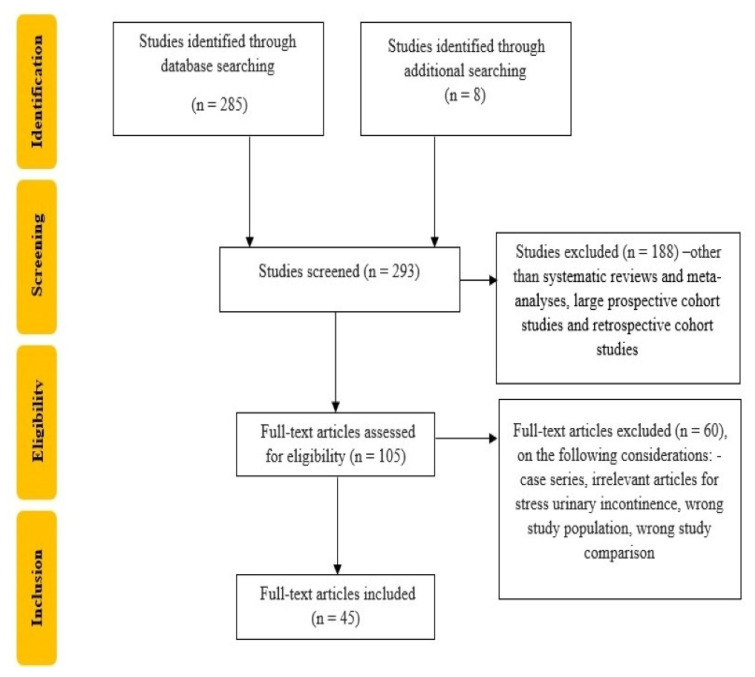
Flowchart of the study selection process.

We conducted a search of the literature limited to the last 10 years. We included systematic reviews and meta-analyses, retrospective cohort studies and large prospective cohort studies in our data synthesis. We hand-searched conference proceedings, journals and reference lists of relevant articles. Case series and case reports were not included in our data synthesis (Figure 1). The following databases were searched during the review process: Medical Literature Analysis and Retrieval System Online (MEDLINE), Web of Science, Pubmed and Cochrane Central Register of Controlled Trials.

A search of the literature was most commonly centered on the three most common surgical methods, including the midurethral sling, Burch colposuspension and autologous pubovaginal sling [Table 1].

This review was registered to PROSPERO with the ID NR. CRD42023409430/10.04.2023.

### 1.2. Data Collection and Analysis

Cochrane Incontinence published eight systematic reviews of randomized controlled trials that analyzed nine surgical interventions used for the treatment of stress urinary incontinence in women. These reviews represent an excellent literature base of evidence for surgical interventions in this domain, published in the last 5 years. The multitude of possible comparisons makes it difficult for both women and health professionals to interpret the current evidence because of the lack of consensus [12,13]. 

All data they collected are systemized into a meta-analysis comparing the clinical benefit of surgical procedures, and they also described adverse events and complications in this type of surgery [1,12,13].

They screened using inclusion criteria, such as randomized controlled trials or quasi-randomized controlled trials, women with urinary stress incontinence or mixed urinary incontinence with predominant symptoms of stress urinary incontinence and comparing two or more surgeries [13,14,15,16].

They included eight surgical methods in the analysis [12,16,17,18,19].

The results were impressive, which was the first extensive attempt to estimate the clinical effects and safety of surgical interventions for the treatment of stress urinary incontinence based on clinical trial evidence.

The conclusions of the study are that, in the short- to medium-term (one year), retropubic MUS, transobturator MUS, traditional sling and open colposuspension seem to be more effective for the treatment of this type of incontinence. Evidence was insufficient to describe the long-term effectiveness and safety of surgical treatments [15,16].

Careful consideration of all surgical options and a better understanding of their associated risks is a must before choosing a different, potentially less effective, non-MUS procedure. Each case must be personalized and adapted to the need and desires of the patient.

A search of the literature was centered on the three most common surgical methods, including the midurethral sling, Burch colposuspension and autologous pubovaginal sling. The reason why the literature is centered on these three techniques is due to the fact that although there is a multitude of complex techniques described, the research groups are very small and inconsistent, and the techniques are non-standardized. Each surgeon can imagine a personalized technique, but this leads to deficient anticipation of the postoperative evolution and to a low safety profile, so we can only take into consideration established and intensively studied techniques for which we have reported results, analyzed techniques and large study groups.

### 1.3. Midurethral Sling

A midurethral sling system is designed to provide a strip of support under the urethra to prevent it from falling during physical activity. It is considered that this procedure has a symptom cure rate of 80–95% and an objective cure rate of 57–92% [15]. 

Midurethral sling, as a surgical procedure for SUI, was the most extensively studied regarding the efficacy and the safety profile, so most of the reviews have the highest evidence-based results on this procedure.

Midurethral sling involves inserting a strip of synthetic mesh through the retropubic space or obturator foramen. Transobturator (TOT) was developed to minimize the potential risk for bladder injuries and is considered to be the safer of the two options because, unlike TVT, it avoids a surgical approach between the pubic bone and the bladder. TOT and TVT are both made of polypropylene mesh [15,19].

In 2009, Cochrane’s review of 12,113 women described the efficacy between the two procedures as similar, with a lower risk of vascular injuries and urinary retention in the transobturator approach. The most recent reviews update reconfirmed the data. Furthermore, they were also described to be more cost-effective. Fusco et al. performed an updated systematic review to compare the efficacy and safety of MUS compared to Burch colposuspension and pubovaginal slings and found that MUS was appreciably superior for overall cure rates. They had a comparable risk of further incontinence surgery and late complications [20]. 

Morling et al. described a study performed on 16,660 women who had undergone a first single procedure with mesh compared with colposuspension, a mesh method that was found to be more effective regarding immediate complications and with low second interventions for relapse [21,22].

In 2017 in an updated systematic review, the advantage of TVT against retropubic urethropexy and autologous fascia sling was reconfirmed [20].

In 2018 a multi-center study compared the tension-free vaginal tape and Burch colposuspension showed a higher rate of continence in the TVT Arm at 2 years [23].

A 2013 Nordic multi-center cohort study demonstrated an objective cure over 90% at 17 years after TVT with only one mesh complication [24].

Regarding mesh safety, concerns about complications such as erosion, pain, dyspareunia and infections were described. These complications can result in disabling symptoms requiring major surgery and can sometimes reduce the quality of life. Additional questions regarding polypropylene carcinogenic properties were raised, but a nationwide Swedish cohort study including over five million women showed no association with increased cancer risk following MUS surgery [25].

In general, the complications after MUS surgery appear to be lower than alternative non-mesh-based SUI surgeries [26].

A Scottish independent review was reported in March 2017 and deduced that a retropubic mesh tape is a good option with good safety and efficacy profile [27].

It is important to know that some of the women will suffer serious complications that can affect their quality of life and are not easy to correct, but in most cases, they will describe an improvement in SUI after treatment by a surgeon with high experience.

In 2022, a single-center prospective study that evaluated the functional outcome at 20 years after the vaginal sling procedure showed that the functional outcomes decline after 20 years post-correction, so the cure rate after 5 years of TOT implantation was 80%, and the follow-up showed that they dramatically decreased over the years. The limitation of this study is that it is a single-center study, and the strengths are that it is one of the few studies that evaluate the patient’s long-term satisfaction, but we all need to evaluate more carefully the long-term outcomes in our patients, not only in 5 years but longer [28].

All of these studies have a general and common idea that, when used in appropriate patients by appropriately trained surgeons, mesh procedures have a good safety and efficacy profile [27]. 

### 1.4. Autologous Pubovaginal Sling

The autologous fascial pubovaginal sling was first described in 1978 and involves collecting a piece of rectus fascia or sometimes tensor fascia lata which is placed under the urethra after making a small vaginal incision. This procedure is described as taking longer operative time because of the dissection and harvesting operative time, higher blood loss involving dissection and postoperative morbidities such as seroma, so nowadays, it is reserved for women with recurrent SUI, severe SUI or previous complications of MUS surgery [29,30].

The pubovaginal sling is also indicated in the case of a urethral fistula or diverticulum repair because of its obstructive effect and concomitant urethral reinforcement [30,31]. 

The low risk of complications, such as erosion due to foreign bodies associated with synthetic mesh, is the principal advantage of this technique.

A contemporary large systematic review from the UK that described comparative the pubovaginal sling with colpourethro suspensions and MUS showed similar success rates regarding efficacy profile [12].

In 2007, a trial called the Stress Incontinence Surgical Efficacy trial made at 24 months from the procedure described that the rate of success was higher for autologous pubovaginal sling compared with colposuspension, although side effects and need for reintervention was higher in the autologous pubovaginal sling [31]. 

Schimpf et al. found in their study that autologous pubovaginal sling was superior, comparing subjective cure rates, to Burch colposuspension; however, MUS was found to be superior to the pubovaginal sling regarding cure rates and had a lower incidence of overactive bladder symptoms [32].

The pubovaginal sling is an effective procedure with high satisfaction rates but with additional morbidity of harvesting of fascia. Concerns regarding mesh-related complications increased interest in the pubovaginal sling technique because of the few pain-related complications and lack of mesh erosions reinterventions. However, the described surgical morbidity and low surgeon expertise may be obstacles to wider use of this procedure [32,33].

### 1.5. Burch Urethropexy

This procedure consists of a suspension of the anterior vaginal wall to the iliopectineal ligament and has been the main surgical procedure for many years and can be performed open or by laparoscopic approach. Complications of this intervention include bladder perforation, overactive bladder syndrome, prolapse and hemorrhagic incidents.

Because of its use for more than 50 years, the Burch procedure has data on long-term outcomes, with cure rates up to 82% at 5- to 10-year follow-up. A large review found similar results, and the continence rates for open Burch procedures were noted to be 85% at 1 year postoperatively and approximately 70% after 5 years [31]. 

Cohrane’s review from 2017, which included almost 5500 women, reviewed 55 trials and demonstrated Burch urethropexy’s continued efficacy, especially for long-term urinary management.

The procedure can also be laparoscopically performed. A study conducted at 2 years postoperation was shown to be as effective as an open Burch procedure, however, with a significantly longer operative time but a shorter length of hospitalization [16,31].

EAU guidelines suggest that any type of approach, open or laparoscopic, has comparable cure rates, and it has to be offered as an alternative when a midurethral sling cannot be considered [33]. 

NICE guidelines suggest that laparoscopic colposuspension should be made only by an experienced laparoscopic surgeon who is part of a multidisciplinary team [34].

Burch and robotic Burch, which are minimally invasive options for treating this condition, have similar outcomes but lack comparison data [35].

The robotic Burch urethropexy was first reported by Francis and colleagues in 2015. The cost of robotic surgery procedures is known to be significant, and the authors propose that a robotic approach in this pathology is particularly useful in patients that benefit from other concomitant robotic surgeries, especially in the pelvic area [36,37].

Another minimally invasive variant of the Burch colposuspension is the “Mini-Incisional Burch”. This procedure was proposed by Lind and colleagues in 2004, with the desire to allow through a smaller incision the same surgical correction. The authors of the study reported a complete cure in 97% of patients at short-term follow-up [35]. 

Due to the intensive use of sling operation, the use of the Burch colposuspension has decreased nowadays but still has a significant role in the treatment of stress incontinence. Specifically, this strategy has to be considered when we have limited space access, intra-abdominal concurrent surgery or contraindications in the use of mesh.

## 2. Discussion

SUI generates a lower quality of life, having an effect on emotional well-being, depression and anxiety, alternating relationship activities, low self-esteem and sometimes associated comorbidities. Given the impact of the clinical manifestation, it also implies an economic impact for both the individual and society, especially in our country, where the patient is much less compliant with intermediate therapies and early investigation of the health problem [38].

Milson and Coyne (2014), in a systematic review of economic costs, report that in multinational studies, the annual estimated cost of disease for UI was billion-wise in five European countries in 2010. In terms of cost efficacy, in recent years, health economics has been a very important area in decision-making because, with their studies, they make plans for the development of medical strategies by considering the costs involved in different medical approaches. Regarding SUI, there are involved high direct economic costs expressed in a decrease in labor productivity that leads to a second economic loss for society in all areas [39]. 

In addition to the economic and social implications, the implications in the sexual sphere are not to be neglected. Due to the pelvic anatomy and the proximity of the urinary tract to the genital tract, sexual problems can occur. Women who suffer from this pathology may associate anxiety with the possibility of losing urine during sexual intercourse. Such a dysfunction involving low predictability of the event and poor control can determine the woman not wanting sexual intercourse or being anxious during it, something that leads to couple and trust problems. Studies on female sexual dysfunction estimate sexual disturbances in 39–45% of sexually active women. Several papers studied the relationship between urinary incontinence and sexuality, and they concluded that urgency symptoms or even urine loss were associated with anxiety, mood disturbances and low quality of life of SUI in the context of sexual life, sometimes leading to depression [40]. 

The pathophysiology of stress urinary incontinence in complex and the unitary action of all pelvic anatomical structures, such as urethral support, vesical neck function and urethral muscles, is important in maintaining balance. Stress incontinence appears when the pressure inside the bladder, as it fills with urine, becomes higher than the strength of the urethra to stay closed [3]. 

Slow-contractile muscle fibers from the urethra generate tension and maintain the closed urethra [3]. The mechanism prevents urine leakage and keeps urethral pressure during stress periods. If the urethra has trauma, such as radiotherapy or surgery, the urethral wall will lose elasticity and secondary closure capability [3].

Perhaps one of the most important aspects of continence control concerns the balance between all of these systems. The anatomical pathogenesis of SUI facilitates a better understanding of the mechanism of SUI and provides new ideas for surgical treatment. There are several theories that explain the dysfunctions in SUI at the level of each system as well as imbalances in the whole system. There are various anatomical factors, but the majority of the studies pay much attention to the joint contraction of the levator ani muscle and the external urethral sphincter. Therefore, in the treatment of SUI, the solution is the repair and reconstruction of the levator ani muscle and external urethral sphincter. The surgical technique should be improved according to findings in anatomical pathogenesis of SUI, and by anatomical basis, we should develop and use more appropriate slings to reduce the impact of surgical operations on pelvic organs [41].

## 3. Conclusions

There are many options for treating SUI in women, the most used are autologous fascial slings, midurethral tapes and colposuspension. Historically, colposuspension was the most used procedure, but now, after years of research, most patients are offered midurethral tape. The new guidelines (EAU, NICE and Cochrane) recommend offering first-choice MUS to women with SUI and no complications as the preferred surgical intervention.

It is important for the surgeon to discuss with the patient the risks and benefits of each intervention, to evaluate their personal needs, social status and how incontinence affects their life. The choice of the technique and the operative moment must be adapted to the patient’s expectations. They must be realistic and as reproducible as possible postoperatively. The role of the surgeon is to mediate the patient’s needs with the surgical possibilities.

MUS remains the most extensively studied treatment with the lowest complication rates, but women should be informed regarding the rare but serious erosions mesh-related complications. Perspectives in the development of new bio-synthetic mesh materials may lead to new surgical techniques with fewer complications.

Careful contemplation of alternative surgical options and a better comprehension of their associated risks and benefits is a must requirement before opting for different, potentially less effective non-MUS procedures.

Increasing concerns regarding mesh-related complications renewed interest in autologous fascial slings because of the lack of complications of mesh erosions, but the potential morbidity after harvesting of fascia makes it less used. By improving the fascia sampling technique and standardizing the insertion procedures, we can obtain better results with minimal complications.

The newest guidelines (EAU guidelines) suggest providing a colposuspension procedure if a midurethral sling cannot be considered.

A better evaluation and acknowledgment of complications after surgery for stress urinary incontinence is imperative. It is important to promote awareness of later complications, and it could be very useful to record them in national databases and registries to generate uniform data for each surgeon to access.

Both at the scientific level and at the level of each patient, there is a clear need to identify personal needs as well as to evaluate the associated risks for good control of the risks and benefits of each intervention.

## Figures and Tables

**Table 1:** Inclusion/Exclusion criteria.

Criteria	Inclusion Criteria	Exclusion Criteria
	Study population female Written in English language Studies made on established techniques. Systematic reviews and meta analyses. Large prospective cohort studies and retrospective cohort studies. Stress urinary incontinence.	Letters to the editors. Case reports. Clinical trials. Studies made on small study groups. Comparison between technical variation of the same procedure. Studies written in any other language then English.

## Data Availability

Not applicable.

## Abbreviations

SUI	Stress urinary incontinence
MUS	Midurethral sling
EAU	European Association of Urology
NICE	National Institute of Health and Care (England)
MUS	Urethral sling
TOT	Transobturator
TVT	Tension-free vaginal tape
